# Are associations between psychosocial stressors and incident lung cancer attributable to smoking?

**DOI:** 10.1371/journal.pone.0218439

**Published:** 2019-06-20

**Authors:** Carolyn E. Behrendt, Candace M. Cosgrove, Norman J. Johnson, Sean F. Altekruse

**Affiliations:** 1 Biostatistics and Epidemiology, Information Sciences, City of Hope National Medical Center, Duarte, California, United States of America; 2 Mortality Research Branch, United States Census Bureau, Suitland, Maryland, United States of America; 3 Surveillance Research Program, Division of Cancer Control and Population Sciences, National Cancer Institute, Rockville, Maryland, United States of America; Brown University, UNITED STATES

## Abstract

**Purpose:**

To learn whether reported associations between major psychosocial stressors and lung cancer are independent of smoking history.

**Methods:**

Subjects were at least 25 years old and without lung cancer at enrollment in the United States Census Bureau’s National Longitudinal Mortality Survey in 1995–2008. Follow-up via Surveillance Epidemiology and End Results and National Death Index continued until lung cancer diagnosis, death, or December 2011. Involuntary unemployment, widowhood, and divorce, stratified by sex, were tested for association with subsequent lung cancer using proportional hazards regression for competing risks. Smoking status, years smoked, cigarettes per day, and years since quitting were imputed when missing.

**Results:**

At enrollment, subjects (n = 100,733, 47.4% male, age 49.1(±15.8) years) included 17.6% current smokers, 23.5% former smokers. Of men and women, respectively, 11.3% and 15.0% were divorced/separated, 2.9% and 11.8% were widowed, and 2.9% and 2.3% were involuntarily unemployed. Ultimately, 667 subjects developed lung cancer; another 10,071 died without lung cancer. Adjusted for age, education, and ancestry, lung cancer was associated with unemployment, widowhood, and divorce/separation in men but not women. Further adjusted for years smoked, cigarettes per day, and years since quitting, none of these associations was significant in either sex.

**Conclusions:**

Once smoking is accounted for, psychosocial stressors in adulthood do not independently promote lung cancer. Given their increased smoking behavior, persons experiencing stressors should be referred to effective alternatives to smoking and to support for smoking cessation.

## Introduction

The risk of lung cancer has been associated with a history of major psychosocial stressors, such as the death of a spouse [1], adverse childhood experiences [2], parental loss in early adulthood [3], stressful workplace [4], and involuntary job changes [5] or unemployment [6]. Marital instability in men (evidenced by having children with more than one woman) is another psychosocial stressor that has been associated with risk of lung cancer [7]. However, the latter studies have not always taken history of smoking into account, leaving open the possibility that psychosocial stress increases the risk of lung cancer simply by promoting smoking. On the societal level, it has been observed that “populations that experience higher levels of stressful events smoke more heavily and eventually experience higher mortality from lung cancer” [8]. Alternatively, psychological stress might interact with smoking to elevate the risk of lung cancer, as depression has been observed to do [9,10].

On the other hand, studies in animal models of lung cancer support various roles for psychosocial stress in promoting lung cancer, particularly adenocarcinoma. In mouse models involving xenografts from lung adenocarcinoma, experimentally-induced chronic social stress (changing box-mates twice a week or repeated social defeat) significantly increases the weight and volume of primary tumor and the number of metastatic nodules in the lung [11, 12]. The physiological stress response is characterized by increased secretion of hypothalamic and pituitary stress hormones, which can trigger and maintain chronic inflammation, known to play various roles in tumor promotion [13]. *In vitro*, norepinephrine and epinephrine (called the stress neurotransmitters) stimulate cell proliferation and migration of various tumor cells, including lung adenocarcinoma [14, 15]. *In vivo*, in a hamster model of lung adenocarcinoma, chronic exposure to epinephrine significantly increases the number of primary tumors [16]. Conversely, in a mouse model of lung adenocarcinoma, stress reduction (via housing in an environment enriched with sensory, physical and social stimulation to residents) results in tumors of lower weight than standard housing does [17].

Epidemiological databases offer opportunities to test specific associations with cancer while controlling for history of smoking. One such resource for cancer epidemiology is the ongoing NLMS-SEER database [18], initially created in 1999 by linking the voluntary National Longitudinal Mortality Survey (NLMS, which includes matching to the National Death Index) to the Surveillance Epidemiology and End Results (SEER) database of mandated cancer registries. The NLMS-SEER was designed “to overcome the absence of individual-level socioeconomic data in cancer registries”, particularly data on educational attainment, income, employment, general health, health insurance, and smoking [18]. Recently, NLMS-SEER data through 1998 were used to explore associations between socioeconomic factors and incidence of solid tumors [18]. According to that study, the age-adjusted incidence of lung cancer was increased among subjects who began follow-up as unemployed-but-looking-for-work (rate ratio: 1.83, 95% CI 1.37–2.44, in men; similarly, 2.09, 1.32–3.31, in women) relative to employed subjects. Also at increased risk of lung cancer were divorced or separated individuals (1.34, 1.10–1.63, in men; 1.83, 1.49–2.25, in women) and widowed women (1.45, 1.19–1.76) but not widowed men (0.96, 0.72–1.29). Similar associations were not detected with solid tumors at other sites (colon/rectum, prostate, female breast, melanoma). These preliminary findings suggest that the psychosocial stress of losing one’s job or marital relationship increases the risk of developing lung cancer. However, the latter study, like others cited above [1, 3, 6, 7], did not control for potential confounding by smoking.

Associations with lung cancer that arise through such confounding are revealed as nonsignificant when the analysis is adjusted for smoking-related variables. However, not all associations with lung cancer are confounded by smoking: recent examples of risk factors that remain significantly associated with lung cancer after adjustment for smoking include central obesity [19] and low socioeconomic status [20]. To investigate whether involuntary unemployment, divorce, and widowhood are associated with incident lung cancer independently of smoking, we analyzed a recent cohort from NLMS-SEER followed through 2011. Our statistical analysis accounted for the competing risk of death without lung cancer diagnosis and the clustering of individuals within NLMS households, as well as years smoked, cigarettes per day, years since smoking cessation, and demographic and socioeconomic covariates. Because most of the preclinical evidence that stress contributes to lung cancer [11,12,14–17] is specific to adenocarcinoma, our analysis also explored whether psychosocial stressors are associated with risk of lung adenocarcinoma specifically.

## Materials and methods

Led by the United States Census Bureau, NLMS-SEER is an ongoing study of the socioeconomic characteristics and incidence of cancers and non-cancer mortality among a complex survey sample weighted to be representative of the American population. The NLMS survey is voluntary; interviewees give informed consent and understand that survey responses are confidential and may be linked for statistical analysis to data from other government databases. The NLMS-SEER database is maintained in a manner that precludes identification of individual subjects. Performed as a secondary analysis of existing, de-identified data, the current study did not require approval by an institutional review board or additional informed consent.

Eligibility criteria for the current study were inclusion in the linked NLMS-SEER database and age 25 to 98 years at enrollment during 1992 through 2008, except in those years (1994, 1997, 2000) when the Tobacco Use Supplement to the Current Population Survey [21] was not routinely administered as part of the NLMS interview. Excluded from analysis were individuals with missing race/ethnicity (hereafter referred to as ancestry), missing sampling weight, or who resided in a county for which sex-specific estimates (for the prevalence of current and ever smoking) were unavailable [22].

The Current Population Survey periodically selects for interview a probability-based complex sample of the non-institutionalized population age 25 and older, drawn from a sampling frame of households in the United States as prepared for the most recent decennial U.S. Census. Ethnic minorities and residents of sparsely populated states are over-sampled. Due to the study’s complex sampling, all analyses require weighting by the survey sampling weights. Of households selected for interview, 96% choose to participate [Norman Johnson, personal communication]. Interviews, in person or by telephone, are conducted with one interviewee per household, who provides socioeconomic and demographic data on him- or herself and on each of the other residents. Thus, NLMS subjects cluster within households.

After the enrollment interview, those NLMS subjects enrolled from counties covered by SEER (cancer registries for all or part of 12 states: California, Connecticut, Georgia, Hawaii, Iowa, Kentucky, Louisiana, Michigan, New Jersey, New Mexico, Utah, Washington) are followed not only for mortality through the National Death Index but also for cancer diagnoses through SEER. In the current study, follow-up continued through 2011. The outcome of primary interest was clinical diagnosis of lung cancer as noted in SEER, with histological subtype recorded by ICD-O-3 morphology code.

All risk factors were assessed at the baseline interview. The hypothesized risk factors for lung cancer, stratified by sex, were marital separation or divorce, widow(er)hood, and being in the labor force but unemployed. Data on the major potential confounding factor, history of active cigarette smoking, were obtained from the Tobacco Use Supplement questionnaire, which defined current smoking as smoking at least 100 cigarettes in one’s life and smoking cigarettes at least some days at the time of interview [21]. From current and former smokers, the Survey collected the number of years smoked, cigarettes smoked per day while a smoker, and years since quitting smoking. Even in years when NLMS interviews routinely included the Tobacco Use Supplement, not every subject was administered or completed the tobacco use survey.

All statistical analyses were performed using SAS software (version 9.4, Cary, NC). To determine how subjects with and without data on smoking history might differ, a multivariable logistic regression model evaluated sex, marital status, employment status, diagnosis of lung cancer during follow-up, and year of enrollment into the study for association with missingness of smoking status, years smoked, cigarettes per day, and/or years since quitting.

To retain all eligible subjects in the current study, missing data related to smoking were multiply imputed using logistic regression for binary variables and linear regression for continuous variables (S1 Appendix). Independent variables used to impute smoking-related variables were primarily demographic and socioeconomic characteristics but also could include the relationship of the survey respondent (interviewee) to the study subject and county-level, sex-specific estimated prevalence of smoking among adult residents and of having quit smoking among ever smokers (S1 Fig) [22]. The latter county prevalence estimates were assigned to individual subjects according to their county of residence, sex, and year of enrollment in NLMS. Subjects who had enrolled in 1992–99 were assigned estimates from 1997–99, and later enrollees were assigned estimates from 2000–03.

The hypothesized risk factors of involuntary unemployment, widow(er)hood, and divorce, stratified by sex, were tested for association with incident lung cancer as follows. Because death without prior diagnosis of lung cancer preempts a diagnosis of lung cancer, current subjects were at risk of competing outcomes. Accordingly, the analysis used Fine and Gray’s [23] proportional hazards model for the subdistribution (subhazard or cumulative incidence function) of a competing risk. Model 1 tested being divorced or widowed, and Model 2 tested being involuntarily unemployed. Models incorporated the NLMS sampling weights and accounted for the clustering of subjects within households.

Preliminary and final versions of each model of lung cancer were adjusted for demographic and socioeconomic covariates other than smoking, if these improved the model’s fit to the observed data. Considered as potential covariates were decade of age, educational attainment, ancestry, geographic region, urban/rural status, type of recent relocation if any, annual family income, occupation, military veteran status, size of household, type of housing, home ownership, self-rated general health, and health insurance status. The final model differed from the preliminary model by being further adjusted for years smoked, number of cigarettes per day, and years since quitting. The level of Type I error per model was maintained below 5% using the Holm-Bonferroni adjustment for multiple hypothesis testing [24].

For the main evaluation of the study’s hypotheses, adjustment for smoking variables was performed using the 5 imputations, with the resulting 5 models averaged into one using SAS PROC MIANALYZE. As secondary analyses, the hypotheses were evaluated under two alternate scenarios: once using only those subjects with no missing data on smoking-related variables and again using the imputations but restricting the event of interest to lung adenocarcinoma.

## Results

After excluding the 5.2% of eligible participants who lacked requisite data (on ancestry, sampling weight, or county-level prevalence of smoking), the study cohort (47.4% male, age 49.1(±15.8) years) included 100,733 participants. They were demographically and socioeconomically diverse: one third (33.8%) of participants were at least age 55 years at enrollment, 31.3% were of non-White or Hispanic ancestry, 28.4% had at least a 4-year college degree, and another 26.0% had taken some college courses (Table 1). Among male and female subjects, respectively, 11.3% and 15.0% were divorced/separated, 2.9% and 11.8% were widowed, and 2.9% and 2.3% were unemployed but looking for work. The baseline interview was usually conducted with the subject (56.7%) or his/her spouse (30.5%); occasionally, the respondent was another member of the household, either a relative (8.9%) or a non-relative (3.9%) (Table 1).

**Table 1:** Characteristics of subjects at enrollment (N = 100,733).

Characteristic	Subjects (N)	Weighted Percentage
**Sex**		
Male	46,918	47.4
Female	53,815	52.6
**Years of Age**		
25–34	21,463	20.4
35–44	25,950	24.4
45–54	21,890	21.5
55–64	13,833	14.7
65–74	9,886	10.4
75–84	6,295	7.0
85 or older	1,416	1.7
**Ancestry**		
Non-Hispanic White	64,168	68.7
Non-Hispanic Black	8,356	9.8
Hispanic, from Puerto Rico or Cuba	1,354	0.8
Hispanic, from Mexico, Central/South America	10,605	8.2
Hispanic, Mexican-American	1,803	1.1
Hispanic, Chicano/White/Other	1,839	0.8
Asian/Pacific Islander	9,115	7.8
Native American/Other Non-White	3,493	2.9
**Nativity**		
United States (Including Territories)	65,679	65.9
Other Country	16,239	14.9
Missing	18,815	19.2
**Area of Residence**		
Urban	81,799	81.4
Rural	18,934	18.6
**Geographic Region**		
New England	6,647	4.8
Mid Atlantic	11,764	11.2
South Atlantic	6,673	10.2
Northeast Central	5,622	5.6
Southeast Central	5,888	5.6
Northwest Central	6,757	4.1
Southwest Central	4,701	5.7
Mountain	10,605	4.6
Pacific	42,076	48.0
**Self-Reported Health**		
Excellent	21,836	21.7
Very Good	25,429	24.9
Good	20,973	20.6
Fair	8,204	8.3
Poor	3,755	3.9
Not Rated	20,536	20.6
**Education**		
High School or Less	47,341	45.6
Some College	25,855	26.0
Bachelor’s Degree	18,211	18.8
Graduate Degree	9,326	9.6
**Employment Status**		
In Labor Force, Working	63,625	61.8
In Labor Force, Absent	2,719	2.6
In Labor Force, Unemployed	2,496	2.6
Not in Labor Force, Disabled	4,282	4.6
Not in Labor Force, Retired/Student/Other	27,532	28.3
Missing	79	0.1
**Military Veteran Status**		
War Veteran	9,370	9.9
Other Military Service	3,036	3.2
No Military Service	88,188	86.7
Missing	139	0.2
**Health Insurance Status**		
Insured	86,980	86.6
Uninsured	13,753	13.4
**Marital Status**		
Married	67,576	64.3
Widowed	7,117	7.6
Divorced or Separated	12,560	13.3
Never Married	13,480	14.8
**Home Ownership**		
Owned	74,003	73.3
Rented	26,730	26.7
**Type of Housing**		
House, Apartment	96,559	95.7
Mobile Home	4,011	4.2
Other	163	0.2
**Persons in Household**		
1	12,743	14.4
2	31,320	34.8
3	19,367	18.6
4 or more	37,303	32.1
**Annual Family Income**		
Less than $50,000	65,658	65.0
$50,000 - $74,999	18,301	17.9
$75,000 or More	16,642	17.0
Not Reported	132	0.1
**Income as a Percentage of Poverty Level**		
At or Below 100%	8,880	8.4
>100%-200%	16,285	15.7
>200%-400%	30,609	29.4
>400%-600%	20,009	20.1
600% or More	22,725	24.5
Not Reported	2,225	1.9
**Recent Relocation**		
Yes, Within or Between Metro Areas	10,500	11.5
Yes, Within or Between Non-Metro Areas	1,342	1.1
Yes, From Non-Metro to Metro Area	503	0.5
Yes, From Metro to Non-Metro Area	450	0.4
Yes, From Abroad	431	0.5
No	87,507	86.1
**Year of Enrollment in NLMS**		
1993	10,858	10.7
1996	13,354	13.2
1999	18,563	18.3
2001–02	15,212	15.0
2003–04	15,795	15.8
2006	11,975	12.0
2007–08	14,976	15.0
**Interviewee’s Relationship to Subject**		
Self, Residing with Relatives	41,768	39.6
Self, Residing without Relatives	15,142	17.1
Spouse	32,073	30.5
Resident Non-relative	3,466	3.9
Resident Child	3,922	4.5
Resident Parent	1,821	1.8
Resident Sibling	902	1.0
Resident Other Relative	1,639	1.6

Prior to the imputation of missing data, status as a current, former, or never smoker was missing for 12.6% of subjects. In addition, the number of years smoked and number of cigarettes per day, respectively, were unreported for 34.1% and 31.8% of subjects who reported ever smoking. Years since quitting smoking was missing for 12.4% of subjects who reported being former smokers. In all, data on smoking-related variables were at least partially incomplete for 25.5% of subjects. Data on smoking were not missing at random: prior to imputation, males, subjects who enrolled in later years, those who were married, divorced or separated, unemployed, absent from work, disabled, or not in the workforce, and those who developed lung cancer during follow-up were likely to lack data on one or more smoking-related variables (S1 Table). Post-imputation, data on smoking-related variables were complete for all subjects. The study cohort included 17.6% current smokers and 23.5% former smokers. Ever-smokers had smoked median 20 (10th-90th percentile range 6–40) cigarettes per day for median 20 (10th-90th percentile range 5–40) years. Among former smokers, time since quitting was median 14.4 (10th-90th percentile range: 2.0–33.0) years.

The study cohort was followed for diagnosis of lung cancer or death from other cause for a median 9.77 (1^st^ to 99^th^ percentile range: 1.18–18.94) years. Ultimately, 667 subjects developed lung cancer; another 10,071 subjects died without developing lung cancer. Histological subtype of lung cancer was recorded as adenocarcinoma (33.4%), squamous cell (21.9%), small cell (11.2%), large cell (2.9%), adenosquamous carcinoma (1.0%), and subtype not specified (25.3%).

All multivariable models that tested unemployment or marital status for association with incident lung cancer were adjusted for age, education, and ancestry; no other socioeconomic covariates were found to improve the fit of these models. Without adjustment for smoking-related variables, the hypothesized associations of lung cancer with divorce/separation, widow(er)hood, and involuntary unemployment were present for men but not for women (Table 2, under the heading Unadjusted for Smoking). In contrast, upon further adjustment for cigarettes per day, years smoked, and years since quitting, none of the latter associations remained significant, in men or in women (Table 2, under the heading Adjusted for Smoking). Associations with established risk factors that characterize history of smoking were as expected: Risk of incident lung cancer was increased by cigarettes per day and by years smoked and was decreased by years since quitting (Table 2).

**Table 2:** Sex-Specific associations between marital status, unemployment, and incident lung cancer.

Multivariable Models Adjusted for Decade of Age, Education, and Ancestry	Hazards Ratio (95% CI) Unadjusted for Smoking	Holm p	Hazards Ratio (95% CI) Adjusted for Smoking	Holm p
Model 1: Testing Marital Status				
**Divorced/Separated**				
** Male**	**2.18 (1.50–3.16)**	<0.001	**1.07 (0.73–1.58)**	NS
** Female**	**1.07 (0.82–1.40)**	NS	**0.91 (0.69–1.19)**	NS
**Widowed**				
** Male**	**1.51 (1.08–2.10)**	0.062	**0.79 (0.56–1.10)**	NS
** Female**	**1.08 (0.78–1.49)**	NS	**0.75 (0.54–1.04)**	NS
Never Married				
Male	1.09 (0.67–1.77)	U	0.75 (0.46–1.21)	U
Female	1.12 (0.69–1.84)	U	0.97 (0.59–1.58)	U
Married				
Male	1.44 (1.19–1.74)	U	0.97 (0.79–1.18)	U
Female	1.00		1.00	
Per Cigarette per Day	——		1.02 (1.01–1.02)	U
Per Year of Smoking	——		1.04 (1.04–1.05)	U
Per Year since Smoking Cessation	——		0.97 (0.96–0.98)	U
Model 2: Testing Unemployment				
**In Labor Force, Unemployed**				
** Male**	**2.62 (1.40–4.90)**	0.014	**1.66 (0.89–3.08)**	NS
** Female**	**0.60 (0.16–2.34)**	NS	**0.55 (0.14–2.15)**	NS
In Labor Force, Employed				
Male	1.27 (0.98–1.65)	U	0.98 (0.76–1.28)	U
Female	1.00		1.00	
Not in Labor Force				
Male	2.07 (1.58–2.71)	U	1.29 (0.97–1.69)	U
Female	1.33 (1.01–1.74)	U	1.25 (0.95–1.63)	U
Per Cigarette per Day	——		1.02 (1.01–1.02)	U
Per Year of Smoking	——		1.04 (1.04–1.05)	U
Per Year since Smoking Cessation	——		0.97 (0.96–0.98)	U

Abbreviations: CI, Confidence interval; NS, Nonsignificant; U, Untested statistically, because the risk factor was not among the pre-specified hypotheses to be evaluated. The reference category for Model 1 is Married Female Neversmokers and for Model 2 is Employed Female Neversmokers.

On secondary analysis, when subjects were restricted to those having complete data on smoking variables (Scenario 1), most (64.3%) incident cases of lung cancer remained available for analysis. As in the main analysis above, the hypothesized associations of divorce, widowerhood, and involuntary unemployment with incident lung cancer were present only in males and disappeared after smoking history was accounted for (S2 Table). Moreover, for each hypothesized association, the Hazards Ratio from the secondary analysis was within the 95% confidence interval around the corresponding Hazards Ratio from the main analysis.

Alternatively, when the outcome of interest was restricted to those lung cancer cases coded in SEER as adenocarcinoma (Scenario 2), only one third (33.4%) of incident cases of lung cancer remained available for analysis. In that case, none of the hypothesized associations was detectable, even in males prior to adjustment for smoking (data not shown).

## Discussion

The preclinical literature [13–17] has suggested that psychosocial stress, mediated by stress hormones and associated chronic inflammation, contributes to the initiation and promotion of lung tumors, particularly adenocarcinoma. In contrast, epidemiological studies to date [1–10] have not resolved whether, in humans, major psychosocial stressors increase the risk of lung cancer independently of smoking.

For the current study, because data on smoking were incomplete or missing in one quarter of subjects and that missingness varied both by lung cancer outcome and by hypothesized risk factors (marital status, employment status), the main analysis was performed after multiple imputation of the missing data. That main analysis and a secondary one limited to subjects with complete data yielded consistent findings: neither divorce, widowhood, nor involuntary unemployment remains significantly associated with risk of lung cancer after adjustment for years smoked, cigarettes per day, and years since smoking cessation.

Another secondary analysis was attempted after restricting lung cancer cases to those coded specifically as adenocarcinoma. However, that restriction resulted in too few lung cancer events to permit detection of any hypothesized associations, even before adjustment for smoking. If nearly all cases of lung cancer that cancer registrars had coded vaguely (mostly as “non-small cell”, “malignant tumor”, or “carcinoma” not otherwise specified) were in fact adenocarcinomas, then the latter analysis could not recognize almost half of its targeted lung cancer cases.

Previous studies provide support for the current finding that major psychosocial stressors are not associated with incident lung cancer after adjustment for smoking. For example, marital status was not significantly associated with death from lung cancer when current or former smoking was taken into account [25]; an association between uppermost quintile of involuntariness of men’s job-changing history and diagnosis of lung cancer became nonsignificant after adjustment for pack-years of smoking [5]; an association between baseline psychological distress per validated instrument and incident lung cancer mortality lost significance after adjustment for smoking and other covariates [26]; and an association between adverse childhood experiences and hospitalization for lung cancer was attenuated by adjustment for ever, former, moderate, and heavy smoking [2]. In addition, a meta-analysis of 12 European cohort studies that took smoking into account detected no association between high job strain and risk of lung cancer [27].

The view that associations of lung cancer with psychosocial stressors are chiefly mediated by cigarette smoking is supported by research that has documented increased smoking behavior in persons who undergo stressful experiences [8]. For example, divorce is associated with higher risk of initiating or resuming cigarette smoking and, for women, of not being able to quit [28]. Unemployment [29] or involuntary retirement [30] increases the odds of smoking and also increases daily cigarette consumption. A linear relationship has been reported between number of adverse childhood experiences and specific smoking behaviors (early age at initiation, ever, current, and heavy smoking) [2] and between total types of stressful life event experienced and current heavy smoking [31].

We acknowledge that a recent case-control study of men [4] has reported that, independently of smoking, cigarette-years, and number of years since quitting, cumulative work-related stress of at least 15 years duration is associated with moderately-increased risk of cancer in 5 of 11 anatomic sites investigated, including the lung. However, that study was retrospective and thus potentially subject to recall bias; as its authors have acknowledged, “over-reporting of past stressful episodes by cases could explain some, or all, of the elevated odds ratios observed” [4].

In contrast to retrospective studies, cohort studies avoid the potential for recall bias. Typically, cohort studies, including the NLMS-SEER, collect data on risk factors at baseline only. Any subsequent change in status, for example, in smoking, marital or employment status, goes unrecorded and thus is unavailable for analysis. As a result, associations with those risk factors may be minimized (biased towards the null). Still, associations with divorce, widowhood, and involuntary unemployment are currently detectable, albeit only in male participants and then only without adjustment for smoking history.

Our study has several limitations. NLMS-SEER subjects cluster within documented households. Smoking and its cessation reportedly “spread” between spouses and siblings [32], but in NLMS, the missingness of smoking data likewise tends to occur within households. For that reason, the smoking status of family members could not be incorporated into current imputation models.

A further limitation is the lack of data on passive smoking (hours per day of exposure to environmental tobacco smoke), which was not collected by the tobacco use questionnaire. Nor could passive smoking be reliably estimated from the smoking status of household members, because the number of cigarettes smoked inside the home while the passive smoker was present is not available. Thus, the current study does not control for exposure to environmental tobacco smoke.

Additional limitations of the current study relate specifically to features of SEER. That system relies on passive reporting by a network of cancer registrars, and as a result, the characterization of tumors’ histological subtype can be incomplete, as it was for one quarter of incident lung cancers in the current cohort. SEER also restricts NLMS-SEER participants to residents of just 12 states, with disproportionate representation of the Pacific region, especially California. Nevertheless, associations between marital or employment status and lung cancer would not be expected to differ significantly between areas inside and outside of SEER. A further limitation of the NLMS-SEER cohort is that ascertainment of cancer cases could be incomplete, if a subject who relocates out of the geographic area monitored by SEER is subsequently diagnosed with cancer. However, incomplete ascertainment of cases would not bias an association with lung cancer unless relocation out of SEER surveillance is associated with one or more risk factors for lung cancer.

In conclusion, the current study indicates that, once smoking behavior is accounted for, major psychosocial stressors in adulthood (ie, unemployment, widowhood, and divorce) do not in themselves promote the risk of lung cancer. From this finding, we conclude that persons who undergo stressful experiences are at increased risk of lung cancer because they are more likely to smoke. To counter this risk, clinicians and public health professionals should counsel persons who suffer major psychosocial stressors against turning to smoking to relieve stress. Referral should be provided to effective alternatives to smoking and, if indicated, to pharmacologic and social support for smoking cessation.

## Supporting information

S1 Appendix Imputation of missing data on smoking. (DOCX)

S1 Fig Imputation of missing data on smoking. (TIF)

S1 Table Independent risk factors for having missing data on smoking history. (DOCX)

S2 Table Sex-specific associations between marital status, unemployment, and incident lung cancer, COMPLETE RECORDS ONLY (NO IMPUTATION). (DOCX)

## References

[1] Martikainen P, Valkonen T. Mortality after the death of a spouse: rates and causes of death in a large Finnish cohort. Am J Public Health. 1996;86:1087–93. 10.2105/ajph.86.8_pt_1.1087 8712266 PMC1380614

[2] Brown DW, Anda RF, Felitti VJ, Edwards VJ, Malarcher AM, Croft JB, et al Adverse childhood experiences are associated with the risk of lung cancer: a prospective cohort study. BMC Public Health. 2010;10:20 10.1186/1471-2458-10-20 20085623 PMC2826284

[3] Kennedy B, Valdimarsdóttir U, Sundström K, Sparén P, Lambe M, Fall K, et al Loss of a parent and the risk of cancer in early life: a nationwide cohort study. Cancer Causes Control. 2014;25:499–506. 10.1007/s10552-014-0352-z 24500176

[4] Blanc-Lapierre A, Rousseau MC, Weiss D, El-Zein M, Siemiatycki J, Parent ME. Lifetime report of perceived stress at work and cancer among men: a case-control study in Montreal, Canada. Prev Med. 2017;96:28–35. 10.1016/j.ypmed.2016.12.004 27923666

[5] Jahn I, Becker U, Jöckel KH, Pohlabeln H. Occupational life course and lung cancer risk in men. Findings from a socio-epidemiological analysis of job-changing histories in a case-control study. Soc Sci Med. 1995;40:961–75. 7792635 10.1016/0277-9536(94)00151-i

[6] Lynge E. Unemployment and cancer: a literature review In: Kogevinas M, Pearce N, Susser M, Boffetta P, editors. Social inequities and cancer. IARC scientific publications no. 138. Lyon, France: International Agency for Research on Cancer; 1997 p. 343–51. 9353675

[7] Li X, Hemminki K. Cancer risks in men who had children with different partners from the Swedish Family-Cancer Database. Eur J Cancer Prev. 2003;12:355–8. 10.1097/01.cej.0000084001.17383.9d 14512798

[8] Colby JP Jr, Linsky AS, Straus MA. Social stress and state-to-state differences in smoking and smoking related mortality in the United States. Soc Sci Med. 1994;38:373–81. 8140464 10.1016/0277-9536(94)90407-3

[9] Knekt P, Raitasalo R, Heliövaara M, Lehtinen V, Pukkala E, Teppo L, et al Elevated lung cancer risk among persons with depressed mood. Am J Epidemiol. 1996;144:1096–103. 10.1093/oxfordjournals.aje.a008887 8956621

[10] Linkins RW, Comstock GW. Depressed mood and development of cancer. Am J Epidemiol. 1990;132:962–72. 10.1093/oxfordjournals.aje.a115739 2239911

[11] Al-Wadei HA, Plummer HK 3rd, Ullah MF, Unger B, Brody JR, Schuller HM. Social stress promotes and γ-aminobutyric acid inhibits tumor growth in mouse models of non-small cell lung cancer. Cancer Prev Res (Phila). 2012;5:189–96. 10.1158/1940-6207.CAPR-11-0177 PMC3320046 21955519

[12] Wu X, Liu BJ, Ji S, Wu JF, Xu CQ, Du YJ, et al Social defeat stress promotes tumor growth and angiogenesis by upregulating vascular endothelial growth factor/extracellular signal-regulated kinase/matrix metalloproteinase signaling in a mouse model of lung carcinoma. Mol Med Rep. 2015;12:1405–12. 10.3892/mmr.2015.3559 25824133

[13] Hänsel A, Hong S, Cámara RJ, von Känel R. Inflammation as a psychophysiological biomarker in chronic psychosocial stress. Neurosci Biobehav Rev. 2010;35:115–21. 10.1016/j.neubiorev.2009.12.012 20026349

[14] Al-Wadei HA, Al-Wadei MH, Masi T, Schuller HM. Chronic exposure to estrogen and the tobacco carcinogen NNK cooperatively modulates nicotinic receptors in small airway epithelial cells. Lung Cancer. 2010;69:33–9. 10.1016/j.lungcan.2009.09.011 19896235

[15] Al-Wadei HA, Al-Wadei MH, Schuller HM. Cooperative regulation of non-small cell lung carcinoma by nicotinic and beta-adrenergic receptors: a novel target for intervention. PLoS One. 2012;7:e29915 10.1371/journal.pone.0029915 22253823 PMC3257239

[16] Schuller HM, Porter B, Riechert A. Beta-adrenergic modulation of NNK-induced lung carcinogenesis in hamsters. J Cancer Res Clin Oncol. 2000;126:624–30. 11079726 10.1007/PL00008474 PMC12165142

[17] Song Y, Gan Y, Wang Q, Meng Z, Li G, Shen Y, et al Enriching the housing environment for mice enhances their NK cell antitumor immunity via sympathetic nerve-dependent regulation of NKG2D and CCR5. Cancer Res. 2017;77:1611–22. 10.1158/0008-5472.CAN-16-2143 28082402

[18] Clegg LX, Reichman ME, Miller BA, Hankey BF, Singh GK, Lin YD, et al Impact of socioeconomic status on cancer incidence and sage at diagnosis: selected findings from the Surveillance, Epidemiology, and End Results National Longitudinal Mortality Study. Cancer Causes Control. 2009;20:417–35. 10.1007/s10552-008-9256-0 19002764 PMC2711979

[19] Yu D, Zheng W, Johansson M, Lan Q, Park Y, White E, et al Overall and central obesity and risk of lung cancer: a pooled analysis. J Natl Cancer Inst. Epub 2018 Mar 6. 10.1093/jnci/djx286 PMC6093439 29518203

[20] Hovanec J, Siemiatycki J, Conway DI, Olsson A, Stücker I, Guida F, et al Lung cancer and socioeconomic status in a pooled analysis of case-control studies. PLoS One. 2018;13(2):e0192999 10.1371/journal.pone.0192999 29462211 PMC5819792

[21] The Tobacco Use Supplement to the Current Population Survey (TUS-CPS). Bethesda, MD: National Cancer Institute, Division of Cancer Control and Population Sciences, Behavioral Research Program; 2018 [cited 2018 May 24]. Available from: https://cancercontrol.cancer.gov/brp/tcrb/tus-cps/.

[22] Small Area Estimates for Cancer Related Measures: Available Estimates for Cancer Risk Factors and Screening Behaviors. Bethesda, MD: National Cancer Institute, Division of Cancer Control and Population Sciences, Surveillance Research Program; n.d. [cited 2018 May 24]. Available from: https://sae.cancer.gov/nhis-brfss/estimates/.

[23] Fine JP, Gray RJ. A proportional hazards model for the subdistribution of a competing risk. J Am Stat Assoc. 1999;94:496–509.

[24] Holm S. A simple sequentially rejective multiple test procedure. Scand J Stat. 1979;6:65–70.

[25] Lewis DR, Clegg LX, Johnson NJ. Lung disease mortality in the United States: the National Longitudinal Mortality Study. Int J Tuberc Lung Dis. 2009;13:1008–14. 19723382 PMC2765862

[26] Hamer M, Chida Y, Molloy GJ. Psychological distress and cancer mortality. J Psychosom Res. 2009;66:255–8. 10.1016/j.jpsychores.2008.11.002 19232239

[27] Heikkilä K, Nyberg ST, Theorell T, Fransson EI, Alfredsson L, Bjorner JB, et al Work stress and risk of cancer: meta-analysis of 5700 incident cancer events in 116,000 European men and women. BMJ. 2013;346:f165 10.1136/bmj.f165 23393080 PMC3567204

[28] Nystedt P. Marital life course events and smoking behaviour in Sweden 1980–2000. Soc Sci Med. 2006;62:1427–42. 10.1016/j.socscimed.2005.08.009 16162388

[29] Arcaya M, Glymour MM, Christakis NA, Kawachi I, Subramanian SV. Individual and spousal unemployment as predictors of smoking and drinking behavior. Soc Sci Med. 2014;110:89–95. 10.1016/j.socscimed.2014.03.034 24727666 PMC4043205

[30] Henkens K, van Solinge H, Gallo WT. Effects of retirement voluntariness on changes in smoking, drinking and physical activity among Dutch older workers. Eur J Public Health. 2008;18:644–9. 10.1093/eurpub/ckn095 18927184 PMC2727140

[31] Bergelt C, Prescott E, Grønbaek M, Koch U, Johansen C. Stressful life events and cancer risk. Br J Cancer. 2006;95:1579–81. 10.1038/sj.bjc.6603471 17106440 PMC2360746

[32] Christakis NA, Fowler JH. The collective dynamics of smoking in a large social network. N Engl J Med. 2008;358:2249–58. 10.1056/NEJMsa0706154 18499567 PMC2822344

