# Supplementary material for: Are associations between psychosocial stressors and incident lung cancer attributable to smoking?
Source: PLoS One. 2019 Jun 20;14(6):e0218439. doi: 10.1371/journal.pone.0218439 (PMC6586400; doi:10.1371/journal.pone.0218439)
Supplement: S1 Appendix — (DOCX) [file pone.0218439.s001.docx]

**S1 Appendix:** **Imputation of Missing Data on Smoking**

Missing data related to smoking were imputed using a series of 5 models, illustrated in S1 Fig and described below.

**S1 Fig.** **Imputation of Missing Data on Smoking.**

To produce the initial set of 5 imputations, logistic regression was used to impute “ever having smoked”. Covariates in that model were, in decreasing order of importance: Self-reported primary ancestry (African, Asian/Pacific Islander, Puerto Rican or Cuban, All Other Hispanic, Native American or Other Non-White, relative to White Non-Hispanic) stratified by sex; Education (Four-year college degree, Some college, High School or less); Military veteran status (yes or no); Decade of age; Self-rated general health (Poor, Fair, Don’t Know/Missing, Good, Very Good, Excellent); County prevalence of ever smoking among adults; Marital status (Divorced/separated, yes or no); Geographic region (8 regional divisions of the country); Birthplace (In the United States versus Elsewhere or place unknown); Relationship of interviewee to the subject (Child, Other non-self, Self without relatives in household, Self with relatives in household); Year of interview; Employment status (In labor force but unemployed, In labor force at work, In labor force but absent, Not in labor force due to disability, Not in labor force for retirement or other reason); Housing (Mobile home versus House or Apartment); Occupation (Operator of precision tools, yes or no); Health insurance (yes or no); Recent relocation (Move to non-metropolitan area versus Other move or no relocation); Rural or urban status; Size of household; and Annual family income (Below versus above $50,000). The model’s c-statistic (where 0.50 represents agreement no better than chance and 1.0 represents perfect agreement) was 0.70.

The next steps in the imputation sequence (S1 Fig) were limited to the records of ever smokers. Working with the 5 imputations from the preceding model, logistic regression was used to generate single imputations of whether the ever smoker had quit smoking. Covariates associated with having quit smoking were, in decreasing order of importance: Decade of age; Education (Graduate degree, College degree, Some college, High School, Less than High School); Marital status (Divorced or separated, Widowed or never married, Married); Home ownership (Owned house, Owned mobile home, Rented); Geographic region; Age at initiation of smoking (Below 16 years, 16-18 years, 19-24 years, 25-30 years, Older than 30 years, Unknown age); Ancestry (Asian/Pacific Islander, Hispanic, All others) stratified by Sex; General health (Excellent, yes or no); Health insurance (yes or no); Managerial or professional occupation (yes or no); Relationship of interviewee to the subject (Child, Other non-self, Self); County’s adult prevalence of having quit smoking; Employment status (In labor force but unemployed versus All others); Size of household, as a continuous variable; Year of interview (Before 1997 versus after). This model’s c-statistic was 0.75.

Next, for all records identified as ever smokers, missing data on “number of years smoked” were singly imputed using linear regression, with the imputed value restricted to between 0 and 80 years. Covariates associated with years smoked were, in decreasing order of importance: Decade of age; Smoking status (current daily, occasional, former smoker), stratified by sex; Education (Less than High School, High School, Up to Two Years of College, Three Years of College, Bachelor’s Degree, Graduate Degree); General Health (Excellent, Poor, Unknown, All others); Ancestry (Mexican, Other Hispanic or Asian/Pacific Islander, Non-Hispanic Black, Non-Hispanic White); Year of interview (Before 2003 vs after); Housing (Owned mobile home, yes or no); Military veteran status (Veteran of World War II or Korean War, Other Veteran, Non-Veteran, Status Not Available); Birthplace (In the United States, yes or no); Geographic region (East Coast, yes or no). This model explained 46.6% of the variation in years smoked.

For those records identified as former smokers, a separate single imputation supplied data on “number of years since quitting”, with the imputed value restricted to between 0 and 74 years. Covariates associated with years since quitting smoking were, in decreasing order of importance: Decade of age; Higher Education (Graduate degree, Three or Four Years of College, Less College or None); General health (Poor, Fair or unknown, Good, Less than Good); Relationship of interviewee to the subject (Sibling/Parent/Other relative, Husband/Child/Non-relative, Self or Wife); Home owner versus renter; Ancestry (African or Asian/Pacific Islander versus All others); Geographic region (Mid-Atlantic or Northeast Central versus All others); County’s prevalence of quitting smoking. This model explained 26.2% of the variation in years since quitting smoking.

For all records identified as an ever-smoker, a final single imputation yielded “number of cigarettes per day (while a smoker)”, with imputed value restricted to between 1 and 95. Covariates in that linear regression model were, in decreasing order of importance: Ancestry (Mexican or Central/South American, Puerto Rican or Cuban, Chicano or Other Hispanic, Non-Hispanic White, Non-Hispanic Black, Asian/Pacific Islander or Native American); Relationship of interviewee to the subject (Spouse, Child, Parent, Sibling/Non-relative, Self); Decade of age; Year of interview; Size of household (One, Two, Three or more residents); General health (Excellent, Very Good, Less than Very Good); County’s prevalence of smoking among adults, as a continuous variable; Education (Four-year college or graduate degree, yes or no); Frequency of smoking (Daily versus Occasional); County’s prevalence of quitting smoking among adult ever smokers, as a continuous variable. Even though each of those variables was highly significant, the model explained only 6.7% of the variation in daily cigarette consumption.
