# Supplementary figures and images for: Are associations between psychosocial stressors and incident lung cancer attributable to smoking?
Source: PLoS One. 2019 Jun 20;14(6):e0218439. doi: 10.1371/journal.pone.0218439 (PMC6586400; doi:10.1371/journal.pone.0218439)

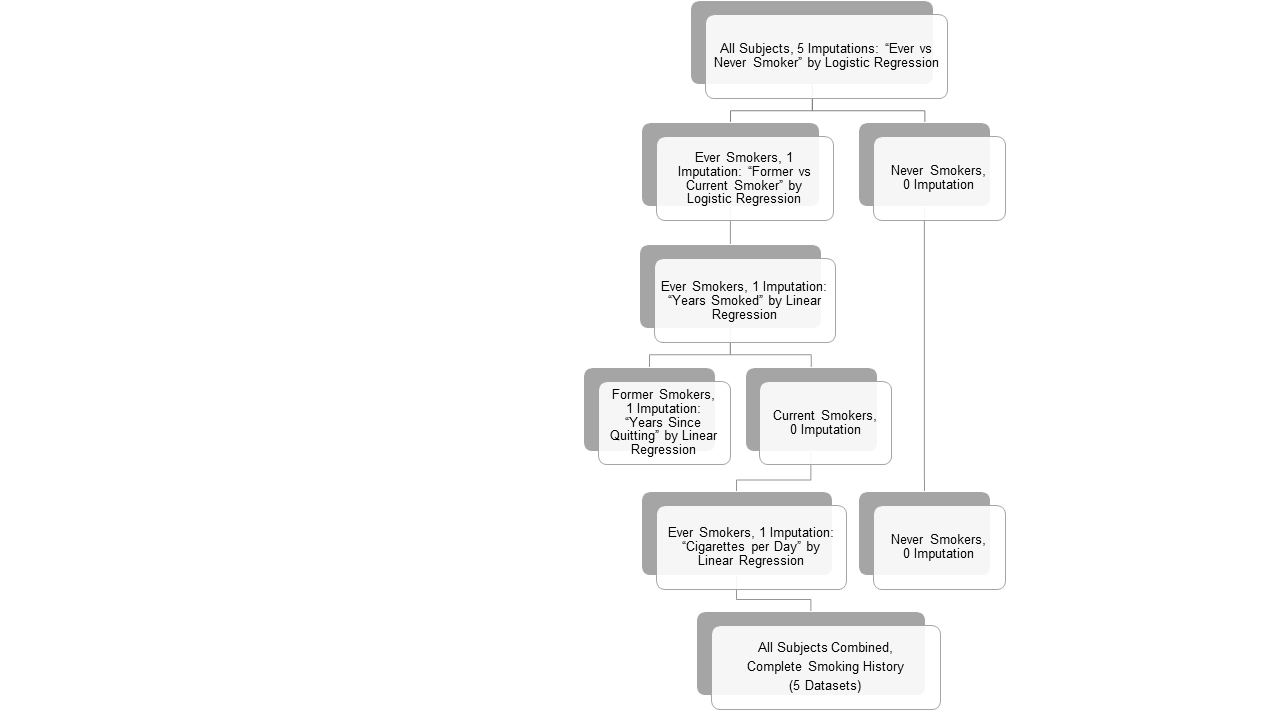

Supplement: S1 Fig — (TIF) [file pone.0218439.s002.tif]
