# Supplementary material for: Are associations between psychosocial stressors and incident lung cancer attributable to smoking?
Source: PLoS One. 2019 Jun 20;14(6):e0218439. doi: 10.1371/journal.pone.0218439 (PMC6586400; doi:10.1371/journal.pone.0218439)
Supplement: S1 Table — (DOCX) [file pone.0218439.s003.docx]

**S1 Table. Independent Risk Factors for Having Missing Data on Smoking History.**

| **Risk Factor** | **Odds Ratio (95% CI)** | **p** |
| --- | --- | --- |
| Year of Enrollment |  |  |
| 1993 | 1.00 |  |
| 1996 | 1.07 (0.99 – 1.16) | 0.10 |
| 1999 | 2.48 (2.31 – 2.66) | <0.0001 |
| 2001 | 2.55 (2.24 – 2.91) | <0.0001 |
| 2002 | 2.27 (2.10 – 2.46) | <0.0001 |
| 2003 | 2.54 (2.35 – 2.74) | <0.0001 |
| 2004 | 1.77 (1.51 – 2.09) | <0.0001 |
| 2006 | 2.65 (2.45 – 2.86) | <0.0001 |
| 2007 | 2.49 (2.31 – 2.69) | <0.0001 |
| 2008 | 3.22 (2.19 – 4.72) | <0.0001 |
| Sex |  |  |
| Male | 1.50 (1.45– 1.55) | <0.0001 |
| Female | 1.00 |  |
| Employment Status |  |  |
| Employed, At Work | 1.00 |  |
| Employed, Absent from Work | 1.16 (1.05 – 1.28) | 0.005 |
| Unemployed | 1.17 (1.05 – 1.31) | 0.004 |
| Disabled | 1.31 (1.20 – 1.42) | <0.0001 |
| Not in the Labor Force | 1.06 (1.02 – 1.10) | 0.007 |
| Marital Status |  |  |
| Married | 1.00 |  |
| Divorced/Separated | 0.98 (0.93 – 1.03) | 0.41 |
| Never Married | 0.92 (0.87 – 0.97) | 0.001 |
| Widowed | 0.83 (0.77 – 0.90) | <0.0001 |

Abbreviations: CI, Confidence interval.

The referent category is Married Women, Employed and at Work, Enrolled in 1993.

For this exploratory analysis, p values are unadjusted for the testing of multiple risk factors.
