# Supplementary material for: Are associations between psychosocial stressors and incident lung cancer attributable to smoking?
Source: PLoS One. 2019 Jun 20;14(6):e0218439. doi: 10.1371/journal.pone.0218439 (PMC6586400; doi:10.1371/journal.pone.0218439)
Supplement: S2 Table — (DOCX) [file pone.0218439.s004.docx]

**S2 Table.** **Sex-Specific Associations between Marital Status, Unemployment, and Incident Lung Cancer, COMPLETE RECORDS ONLY (NO IMPUTATION OF SMOKING VARIABLES)**

| **Multivariable Models Adjusted for Decade of Age, Education, and Ancestry** | **Hazards Ratio (95% CI) Unadjusted for Smoking** | **Holm p** | **Hazards Ratio (95% CI) Adjusted for Smoking** | **Holm p** |
| --- | --- | --- | --- | --- |
| Model 1: Testing Marital Status |  |  |  |  |
| **Divorced/Separated** |  |  |  |  |
| **Male** | **3.00 (1.99 – 4.50)** | <0.001 | **1.12 (0.72 – 1.74)** | NS |
| **Female** | **0.92 (0.66 – 1.29)** | NS | **0.72 (0.52 – 1.01)** | NS |
| **Widowed** |  |  |  |  |
| **Male** | **1.97 (1.37 – 2.84)** | 0.002 | **0.85 (0.58 – 1.23)** | NS |
| **Female** | **1.41 (1.00 – 1.99)** | NS | **0.88 (0.62 – 1.26)** | NS |
| Never Married |  |  |  |  |
| Male | 1.21 (0.69 – 2.13) | U | 0.76 (0.44 – 1.33) | U |
| Female | 1.22 (0.70 – 2.11) | U | 1.02 (0.59 – 1.76) | U |
| Married |  |  |  |  |
| Male | 1.46 (1.16 – 1.84) | U | 0.84 (0.66 – 1.08) | U |
| Female | 1.00 |  | 1.00 |  |
| Per Cigarette per Day | ----- |  | 1.02 (1.01 – 1.02) | U |
| Per Year of Smoking | ----- |  | 1.05 (1.05 – 1.06) | U |
| Per Year since Smoking Cessation | ----- |  | 0.98 (0.97 – 0.99) | U |
| Model 2: Testing Unemployment |  |  |  |  |
| **In Labor Force, Unemployed** |  |  |  |  |
| **Male** | **2.36 (1.10 – 5.05)** | 0.011 | **1.35 (0.64 – 2.87)** | NS |
| **Female** | **0.75 (0.19 – 2.91)** | NS | **0.62 (0.16 – 2.43)** | NS |
| In Labor Force, Employed |  |  |  |  |
| Male | 1.06 (0.78 – 1.45) | U | 0.78 (0.57 – 1.06) | U |
| Female | 1.00 |  | 1.00 |  |
| Not in Labor Force |  |  |  |  |
| Male | 2.27 (1.67 – 3.08) | U | 1.18 (0.86 – 1.63) | U |
| Female | 1.16 (0.85 – 1.59) | U | 1.09 (0.80 – 1.50) | U |
| Per Cigarette per Day | ----- |  | 1.02 (1.01 – 1.02) | U |
| Per Year of Smoking | ----- |  | 1.05 (1.05 – 1.06) | U |
| Per Year since Smoking Cessation | ----- |  | 0.98 (0.97 – 1.00) | U |

Abbreviations: CI, Confidence interval; NS, Nonsignificant; U, Untested statistically, because the risk factor was not among the pre-specified hypotheses to be evaluated.
